# The reliability of the Bad Sobernheim Stress Questionnaire (BSSQbrace) in adolescents with scoliosis during brace treatment

**DOI:** 10.1186/1748-7161-1-22

**Published:** 2006-12-19

**Authors:** Christine Botens-Helmus, Rolf Klein, Carola Stephan

**Affiliations:** 1 Asklepios Katharina Schroth Spinal Deformities Rehabilitation Centre, Korczakstr. 2, D-55566 Bad Sobernheim, Germany

## Abstract

**Background:**

A new instrument to assess stress scoliosis patients have whilst wearing their brace has been developed. Aim of this study was to test the reliability of this new instrument.

**Methods:**

Eight questions are provided focussing on this topic only, including two questions to test the credibility. A max. score of 24 can be achieved (from 0 for most stress to 24 for least stress). We have proposed a subdivision of the score values as follows: 0–8 (strong stress), 9–16 (medium stress) and 17–24 (little stress).

85 patients were invited to take part in this study and to complete the BSSQbrace questionnaire twice, once at the first presentation and a second after a further three days.

62 patients with an average age of 14,5 years and an average Cobb angle of 40° returned their fully completed questionnaires.

**Results:**

The average stress value was 12,5/24 at the first measurement and 12,4/24 at the second measurement. Ceiling value was 23; floor value 2.

The average stress value was 12,5 / 24 at the first measurement and 12,4 / 24 at the second measurement (from 0 for most stress to 24 for least stress). Ceiling value was 23; floor value 2.  There was a correlation of 0,88 (Intraclass Correlation Coefficient) between the values of the two measurements. Cronbach alpha was 0, 97.

**Conclusion:**

The BSSQbrace questionnaire is reliable with good internal consistency and reproducibility. It can be used to measure the coping strategies a patient uses and the impairment a patient feels to have, whilst wearing a brace.

## Background

Quality of life seems to be an important issue for patients with scoliosis. Frequently the SF-36 questionnaire was used to assess health related quality of life (HRQL), which has to be regarded as a general tool already used for quite a variety of diagnoses. In a study related to scoliosis it has already been used alongside the two other questionnaires [1]: Women with idiopathic scoliosis were questioned with the help of an age adapted set of questionnaires containing questions referring to the health related quality of life (SF-36, BFW, STAIK). The results were compared to the norm values and examined in uni- and multivariate procedures (MANOVA) in order to find out whether age or Cobb angle have an impact on quality of life issues. In comparison to the norm random sample, the adolescent female scoliosis population showed a less positive point of view towards life and were subject to depression. Adult patients with scoliosis have shown a reduced HRQL both in the psychological and in the physical field (SF-36). The results were largely independent of Cobb angle or patients' age. The results of the study indicated that idiopathic scoliosis in children, adolescents and adults has to be regarded as a risk factor for the impairment of health related quality of life.

The SRS-22 questionnaire [2-5] has been developed as a tool aiming specifically at spinal deformities. It has been compared to the SF-36 recently [5]. The results of this study show that SRS-22 Mental Health and Pain domain scores can be accurately calculated from compiling SF-36 domain scores. SRS-22 Function scores can be fairly well predicted from correlated SF-36 domain scores. Self-Image and Management Satisfaction/Dissatisfaction domain scores cannot be approximated from SF-36 domain scores.

The SRS-22 HRQL questionnaire is reliable with internal consistency and reproduction comparable to SF-36. In addition, it demonstrated concurrent validity when compared to SF-36. It is shorter and more focused on the health issues related to idiopathic scoliosis than SF-36 [2].

However, the SRS-22 takes more than 20 min. for completion and not all questions seem suitable for patients treated conservatively. Our aim was to establish a short and concise questionnaire related to psychological issues of scoliosis patients, which can be used also in our out-patient practice. Therefore a new instrument has been developed by two physicians and two psychologists working at our centre, to assess the psychological stress scoliosis patients have because of their deformity first [6]. This instrument is called the Bad Sobernheim Stress Questionnaire (BSSQ). 8 questions are provided focussing on this topic. The response to each question is scored 0 (most stress) to 3 (least stress). A max. score values of 24 can be achieved.

The following items have been provided:

1. I feel conspicuous by the appearance of my back.

2. I find it hard to show my back in public.

3. I feel embarrassed in situations, in which other people can see my naked back.

*4. I don't feel embarrassed showing my back*.

5. I try not to get too close to other people to avoid that they become aware of my scoliosis.

6. When deciding what kind of clothes to wear or how to wear my hair, I take care my back is hidden.

*7. Scoliosis is a part of me, people have to accept me the way I am*.

8. Because of the scoliosis I avoid activities/hobbies, which otherwise I would love to do.

Plausibility of the results was assessed from consistent responses to the two most plausible questions (Questions 4 and 7). In order to be credible, the ratings of these questions should not differ more than 1 point to the reference questions (Questions 3 and 5 respectively).

We have proposed a subdivision of the score values as follows: 0–8 (high stress), 9–16 (medium stress) and 17–24 (little stress). The BSSQ was shown to have a good recurrence (r = 0,95) and a sufficient criterion validity (r = 0,78), however a Cronbach α of 0,7 has not been reached [6] and this might have been because of the small number of items [7].

**Figure 1:**
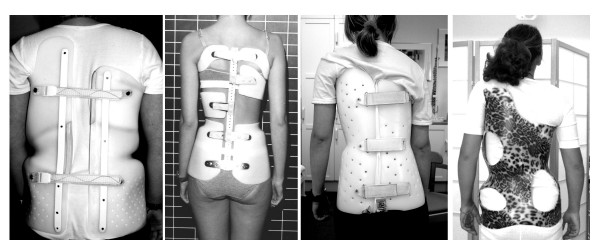
Braces in use for correction of scoliosis.

Quality of life is not only impaired by the deformity or its consequences. Also conservative treatment may contribute to a decreased quality of life. The impact of the treatment procedures, especially with respect to brace treatment (Fig. 1.) on quality of life has not been investigated with special questionnaires until 2005, when the first attempts where undertaken to focus on the problems adolescents might have because of brace wearing [8-10].

Derived from the BSSQ, a questionnaire was developed to estimate the psychological stress adolescent scoliosis patients have because of the brace they wear. This BSSQbrace questionnaire has already been tested previously [8,9]. However the concept of plausible questions has not been applied in the BSSQbrace questionnaire.

As reported in a previous study in brace the patients seem to have more stress than just because of their deformity. The in-brace values of the BSSQbrace showed to be lower than the out-brace values related to deformity [9]. Therefore it seems reasonable to investigate the stress the patients have whilst wearing their brace. As a first step the reiteration of the BSSQbrace should be subject of investigation and this is the purpose of the study presented here.

## Methods

After the preliminary tests [8] a group of 20 adolescents under brace treatment were asked about their opinion as to whether they found the questions reasonably describing their condition. Two patients advised us to change a few words so adolescents would understand the questionnaire better. The final German version of this questionnaire which has been used for the study described in this paper (Additional file 1.), has been translated into English by a native speaker (Additional file 2.).

85 Patients under brace treatment, admitted for in-patient rehabilitation at our centre have been asked to take part in this study and to complete the BSSQbrace questionnaire twice, once at the first presentation and a second after a further three days. 62 (55 females and 7 males) patients with an average age of 14,5 years (SD 1,66) and an average Cobb angle of 40° (SD 20,6), average Cobb angle in brace 27,7° (SD 14,8) agreed to take part and returned their fully completed questionnaires once at the start of in-patient rehabilitation and once again after a further three days.

Intraclass correlation was performed to calculate intraclass correlation coefficient (ICC) using Winstat^® ^Software and Cronbach-alpha was calculated by hand for this statistical procedure is not available in the software package used at our centre.

## Results

The average stress value was 12,5/24 (SD 5,6) at the first measurement and 12,4/24 (SD 5,6) at the second measurement (from 0 for most stress to 24 for least stress). Ceiling value was 23; floor value 2.

The average value of the individual questions (possible range 0 – 3) varied between 0,98 (Question 6) and 2,54 (Question 8). 'The floor value of 0 and the ceiling value of 3 was recorded for each of the 8 questions.

### Reliability

There was correlation of 0,88 between the values of the two measurements which shows the BSSQbrace questionnaire to have a sufficient reliability.

### Internal consistency

Cronbach alpha was 0, 97 which means the internal consistency is high (> 0,7 has to be regarded as sufficient [10]).

17 of the 62 (27%) patients according to the stress rating adjectives of this questionnaire had "strong" stress (score 0 – 8) whist wearing the brace, 31 (50%) rated their stress level as "medium" (score 9 – 16) and 14 (23%) rated their stress level as "little" (score 17 – 24).

20 patients at the time the study was performed wore a brace from our centre and had an average score of 14,5, whilst the 42 patients with braces from elsewhere had an average score of 11,6. The differences of the score values, however were not significant.

30 patients had a thoracic curve pattern (score 11,9 and 12 respectively), 13 patients had a lumbar/thoracolumbar curve pattern (score 12,3 and 13,3 respectively) and 19 had a double major curve pattern (score 13,6 and 12,4 respectively). The differences between the samples were again, not significant.

## Discussion

AIS is considered a possible social problem and furthermore brace treatment may influence the quality of life of the adolescents. There is also an increased parental concern mainly about future pain and disability as an adult [11]. Cosmetic/aesthetic results have also been an important factor to consider in the treatment of adolescent patients with scoliosis [12,13]. AIS and bracing are not associated directly with pain but they can cause discomfort and may influence the patient's daily life in a negative and disturbing way. [10].

Braces in Adolescent Idiopathic Scoliosis (AIS) treatment seem to produce stress [14-17], however there is controversy whether health related quality of life of brace treated scoliotics are affected negatively [1,16,19,20]. AIS has to be regarded as a chronic condition that affects the body configuration of the adolescent, consequently leading to alterations in lifestyle. The impact of the brace to the self and body image of the adolescent is reported as the main contributory factor for stress production [21-29].

The BSSQ questionnaire version for the estimation of deformity related stress had a good reliability, sufficient criterion validity, however a poor internal consistency [6]. The BSSQbrace Questionnaire which is derived from the original BSSQ with a similar structure has good internal consistency and reproducibility. The good internal consistency of the BSSQbrace may be due to the fact, that most of the patients with braces would describe themselves as being impaired by the brace. This also explains why in this study with the BSSQbrace questionnaire, contrary to the BSSQ (deformity) study [6], there was no ceiling effect. The visible deformity can vary greatly and can depend on the Cobb angle [6]. This alone leads to a wide fluctuation of score values. Additionally, patients with scoliosis tend to underestimate their deformity to a certain degree, which might explain the ceiling effect of the BSSQ while the BSSQbrace questionnaire score values seem to be distributed nearly normal.

The in-brace values of the BSSQbrace showed to be lower than the out-brace values related to deformity [9]. This is also supported by the fact that in a subset of braced patients, rating their deformity related stress level (n = 67, Cobb angle 41°) from a study published recently [30], the score values have been higher (BSSQ score value = 20) than in this study with patients rating their stress level in the brace (BSSQbrace score value = 12,5).

The BrQ (Brace Questionnaire)[10], a questionnaire with 8 domains and 34 questions is a reliable and valid means for the assessment of quality of life for patients wearing a brace, however, it needs more time and concentration to be completed and therefore doesn't seem to be useful in the environment of an out-patients practice. For scientific investigations of certain patient groups the BrQ delivers much more information and therefore we would suggest in using the BSSQbrace for the daily routine where information has to be gained fast and to use the BrQ to answer scientific questions arising from daily practice.

There was a tendency for higher BSSQbrace values in patients braced at our centre. This could be due to the fact that the psychological approach used at this centre is more convenient during patient investigation, brace prescription and adjustment than in centres where the numbers of patients being braced are much lower. However, further studies on this topic seem necessary. Therefore, however, the psychological approach to the patient has to be standardized and described in more detail.

As a next step studies are planned to compare this questionnaire to the related domain of a validated health related quality of life tool and to gather data to evaluate the response to change.

## Conclusion

In a series of AIS brace patients the BSSQbrace questionnaire has good score distribution, and is internally consistent and reproducibile. It can be used to measure the coping strategies a patient uses and the impairment a patient feels to have, whilst wearing a brace. Further investigations are needed in order to get a deeper view on the impairment scoliosis patients have during the time of brace treatment.

## Competing interests

The author(s) declare that they have no competing interests.

## Authors' contributions

CBH was responsible for planning of the study, subject acquisition, data analysis and interpretation and the content of the manuscript.

RK contributed in subject and data acquisition.

CS was responsible for the completion of the database

## Supplementary Material

Additional File 1 BSSQbrace original German version Click here for file

Additional File 2 BSSQbrace English version (reliability not tested) Click here for file
