# Supplementary material for: The reliability of the Bad Sobernheim Stress Questionnaire (BSSQbrace) in adolescents with scoliosis during brace treatment
Source: Scoliosis. 2006 Dec 19;1:22. doi: 10.1186/1748-7161-1-22 (PMC1764899; doi:10.1186/1748-7161-1-22)
Supplement: Additional File 1 — BSSQbrace original German version [file 1748-7161-1-22-S1.PDF]

Bad Sobernheimer Skoliose-Fragebogen zur Lebensqualität BSSQK

BSSQ (mit Korsett)

Nachname:............................. Vorname:................................ Pat.Nr.:.....................

Diese Fragen beziehen sich auf Deine Selbstbeurteilung **im Korsett**, also wenn Du das Korsett trägst. Wir bitten Dich, diesen Fragebogen konzentriert und wahrheitsgemäß auszufüllen. Nach Auswertung dieses Fragebogens ist es uns besser möglich die durch ein Korsett hervorgerufenen Belastungen abzuschätzen und Hinweise zur weiteren Behandlung zu geben.

1. Ich fühle mich durch das Aussehen meines Körpers im Korsett beeinträchtigt	- trifft voll und ganz zu<br>- trifft in etwa zu<br>- trifft kaum zu<br>- trifft überhaupt nicht zu
2. Es fällt mir schwer, mich im Korsett offen zu zeigen	- trifft voll und ganz zu<br>- trifft in etwa zu<br>- trifft kaum zu<br>- trifft überhaupt nicht zu
3. Mir sind Situationen unangenehm, in denen andere mein Korsett sehen können	- trifft voll und ganz zu<br>- trifft in etwa zu<br>- trifft kaum zu<br>- trifft überhaupt nicht zu
4. Mein Korsett zu zeigen macht mir nichts aus	- trifft voll und ganz zu<br>- trifft in etwa zu<br>- trifft kaum zu<br>- trifft überhaupt nicht zu
5. Ich vermeide Körperkontakt, um zu verhindern, dass andere mein Korsett bemerken	- trifft voll und ganz zu<br>- trifft in etwa zu<br>- trifft kaum zu<br>- trifft überhaupt nicht zu
6. Ich achte bei der Wahl meiner Kleider oder durch das Tragen langer Haare darauf, mein Korsett zu verdecken	- trifft voll und ganz zu<br>- trifft in etwa zu<br>- trifft kaum zu<br>- trifft überhaupt nicht zu
7. Es macht mir nichts aus, mein Korsett den Personen meines näheren Umfeldes (Eltern, Freunde, Schulkameraden) zu zeigen.	- trifft voll und ganz zu<br>- trifft in etwa zu<br>- trifft kaum zu<br>- trifft überhaupt nicht zu
8. Wegen des Korsetts verzichte ich auf Freizeitaktivitäten/Hobbys, die mir eigentlich am Herzen liegen	- trifft voll und ganz zu<br>- trifft in etwa zu<br>- trifft kaum zu<br>- trifft überhaupt nicht zu

© 2006, Dr. med. Hans-Rudolf Weß
Asklepios Katharina-Schroth-Klinik
