# Supplementary material for: The reliability of the Bad Sobernheim Stress Questionnaire (BSSQbrace) in adolescents with scoliosis during brace treatment
Source: Scoliosis. 2006 Dec 19;1:22. doi: 10.1186/1748-7161-1-22 (PMC1764899; doi:10.1186/1748-7161-1-22)
Supplement: Additional File 2 — BSSQbrace English version (reliability not tested) [file 1748-7161-1-22-S2.PDF]

# Bad Sobernheim Stress Questionnaire BSSQ (brace)

Last name: Surname: Number of patient:

These questions are about how you feel whilst wearing a brace.
Please read this questionnaire carefully and answer truthfully. After the evaluation of this questionnaire it makes it easier for us to estimate, or recognize any psychological stress caused by your brace and give advice for further treatment.

1. I feel uncomfortable by the appearance of my body in the brace
- completely true
- nearly true
- hardly true
- not true at all

2. It is hard for me to be open with my brace.
- completely true
- nearly true
- hardly true
- not true at all

3. I feel uncomfortable in situations where other people can see my brace.
- completely true
- nearly true
- hardly true
- not true at all

4. I don’t feel embarrassed when people see my brace
- completely true
- nearly true
- hardly true
- not true at all

5 I avoid body contact so that no-one knows that I wear a brace.
- completely true
- nearly true
- hardly true
- not true at all

6. When deciding what kind of clothes to wear or how to wear my hair, I try to make sure my brace is hidden
- completely true
- nearly true
- hardly true
- not true at all

7. I don´t feel embarrassed to show my brace to people close to me (parents, friends and schoolfriends)
- completely true
- nearly true
- hardly true
- not true at all

8. Because of my brace I avoid activities/hobbies, which otherwise I love to do
- completely true
- nearly true
- hardly true
- not true at all

© 2006, Dr. med. Hans-Rudolf Weß
Asklepios Katharina-Schroth-Klinik
